# Investigation of esthetic evaluation and its influencing factors for a tunnel portal based on dynamic vision

**DOI:** 10.1371/journal.pone.0238762

**Published:** 2020-09-23

**Authors:** Fei Ye, Enjie Su, Yanchun Wei, Changxin Xu, Xing Liang

**Affiliations:** School of Highway, Chang'an University, Xi'an, Shanxi, China; Tongii University, CHINA

## Abstract

With the development of modern cities, roads, and landscapes, it is becoming increasingly important for infrastructure such as tunnels to provide an esthetically pleasing experience. In this respect, it is necessary to conduct studies that consider the esthetic design of tunnel portals using esthetics research. Regarding the esthetic evaluation of tunnel portals, this paper fully considers the dynamic visual effect from the driver’s perspective. This study combines the use of Blender, SpeedTree Modeler Cinema, Adobe Photoshop CS6, and other software for secondary development. These programs are connected to the driving simulation platform Euro Truck Simulator 2 (which is equipped with a driving simulator) to construct a set of driving simulation tests that enable the esthetic evaluation of a tunnel portal. The Banlun Tunnel on the Funing–Longliu Expressway in Yunnan Province, China, is used as a case study, and four impact factors that vary significantly in esthetic design are included: the linearity, color, greening and texture of the portal. Using an orthogonal experimental design, the influence of the esthetic degree was simulated and evaluated, and the order of sensitivity to esthetic factors of a headwall tunnel portal was sequentially determined as follows: the portal texture exerts the maximum impact on the beauty degree of the headwall portal, followed by the portal greening and the portal color, while the portal linearity exerts the minimum impact. The results show that the developed driving simulation test system can be used to determine the sensitivity of esthetic factors for a tunnel portal and obtain an optimal collocation of esthetic factors on different levels; hence, it provides feedback for use in designing the optimum esthetic tunnel portal. This test system can be used as a reference when conducting future evaluations and studies on tunnel portal esthetics.

## 1. Introduction

With continual improvements in living standards, traffic facilities such as tunnels are not only expected to be functional but also required to provide a sense of comfort and to be esthetically pleasing [1, 2]. Tunnel esthetics are thus the focus of certain studies, as are methods for determining the evaluation of esthetics. For example, a theoretical basis for esthetic evaluation has been proposed [3–8], and a series of esthetic evaluation practices have been implemented [9–15]. Carlier and Moran proposed an approach for characterizing a proposed greenway corridor landscape that focused on habitat composition and ecosystem connectivity, and the results of this study contributed to the realization of European greenways as green infrastructure and true sustainable projects [16]. In addition, Yang used the theory of polybasic mathematics to construct scenic beauty estimation models, and their results indicated that the main factors influencing the quality of a forest landscape are the green space ratio, canopy coverage, quantity of plant color, contrast between the colors, composition of plant growth forms, and wild-like landscape degree [17]. Furthermore, Sarnowski et al. used the visual landscape attraction (VLA) evaluation method to evaluate the potential of a greenway in a glacial area in North Poland and concluded that the VLA method can be used to facilitate the assignment of greenways in other areas [18].

In recent years, with the rapid development of modern technology, some researchers have also employed 3D visualization, virtual simulations, and other new technologies when conducting esthetic evaluations [19–25]. For example, Martinez et al. researched and developed a model that integrated and evaluated subjective and objective landscape factors based on a GIS platform and then introduced public participation according to European landscape convention (ELC) theory. They subsequently evaluated the persistence of landscapes in the Mediterranean region through “objective, objective-weighted, and weighted” methods [26]. Mori et al. developed a driving simulator virtual evaluation system for road space (VERS III) supported by 3D visualization technology and conducted driving simulation tests; the results realized the improvement of tunnel portals and an evaluation of the cladding pad for the tunnel [27]. In 2014, Jiaojiao et al. employed enhanced thematic mapper (ETM) remote-sensing images of Guangzhou to propose a synthetic index method for creating a weighted stack of standardized environmental factors. The study concluded that the ecological environment in the urban center of Guangzhou was generally poor, although it was relatively better in the north and northeast of the city [28]. In addition, Pang et al. developed the landscape simulation and ecological assessment (LEcA) tool to analyze synergies and trade-offs among five ecosystem services. The LEcA tool shows great potential for evaluating the impacts of alternative policies used in land zoning and forest management with respect to forest ecosystem services, and it can be used to assess the consequences of forest management strategies related to renewable energy and conservation policies [29]. Furthermore, Ahlstrom et al. invited 30 young male drivers to participate in a driving simulator experiment to compare the onset of driver sleepiness between two road environments. The results indicated that an increased task demand is more significant than a stimulating visual environment in causing driver fatigue [30]. Chen et al. studied the influence of a decorated tunnel sidewall on driver brain activities using fMRI. The results indicated that the presence of a decorated sidewall provides drivers with better spatial and speed perception and could help reduce accidents associated with speed judgment [31].

However, although numerous studies on esthetic evaluation methods have been conducted, these studies have mainly focused on urban landscapes and highway landscapes, while few have studied esthetic evaluation methods with respect to tunnel portals. Therefore, certain problems need to be clarified with respect to esthetic evaluation methods used for tunnel portals. For example, the esthetic evaluation methods used are mostly qualitative, and there is a lack of qualitative evaluation methods. In addition, existing methods are unable to implement guidance and feedback for esthetic design. Furthermore, in esthetic evaluations, few people have connected the landscape and the dynamic visual features of drivers. Sample evaluation is usually conducted through the use of pictures, which lack a sense of reality and space. In addition, the subjects of esthetic evaluations are mostly experts, which limits the selection of a design scheme to the perceptions of a single population and is inconsistent with the function of a tunnel as an infrastructure that serves the greater public.

In view of these shortcomings, this study draws on evaluation methods used in highway esthetics research (such as the analytic hierarchy process and fuzzy mathematics) and uses a combination of software that includes Blender, SpeedTree Modeler Cinema (STMC), and Adobe Photoshop CS6 (Ps) based on the driving simulation platform Euro Truck Simulator 2 (ETS2) for secondary development. By incorporating a driving simulator, a set of driving simulation test systems are constructed to enable the esthetic evaluation of tunnel portals. The test system is also used to evaluate the esthetic design of the Banlun Tunnel portal on the Funing–Longliu Expressway in Yunnan Province, China. During the evaluation, experiments are designed as orthogonal tests, and members of the general public are introduced as subjects to evaluate and study the esthetic design scheme of the tunnel portals and its impact factor. Therefore, the results of this study can be used as a reference for evaluating and studying the esthetic design of tunnels.

## 2. Development of the driving simulation test system

The esthetic subjects of highway tunnel portal esthetics are mainly staff and passengers who are in a state of motion relative to the tunnel portals. Under dynamic vision, the esthetic subject's response to the accepted visual information becomes delayed or even dull, and the gaze is gradually reduced. As the speed of the car increases, the psychological tension of the esthetic subject will also increase. Based on the dynamic visual characteristics, we independently developed a set of driving simulation systems to restore the driving scene of tunnel portals and increase the reliability of the esthetic evaluation of tunnel portals.

**Fig 1:**
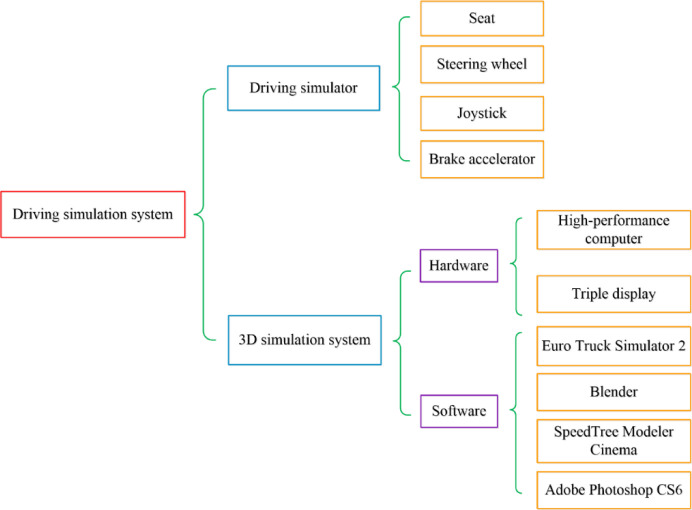
Structural map of the driving simulation system.

**Fig 2:**
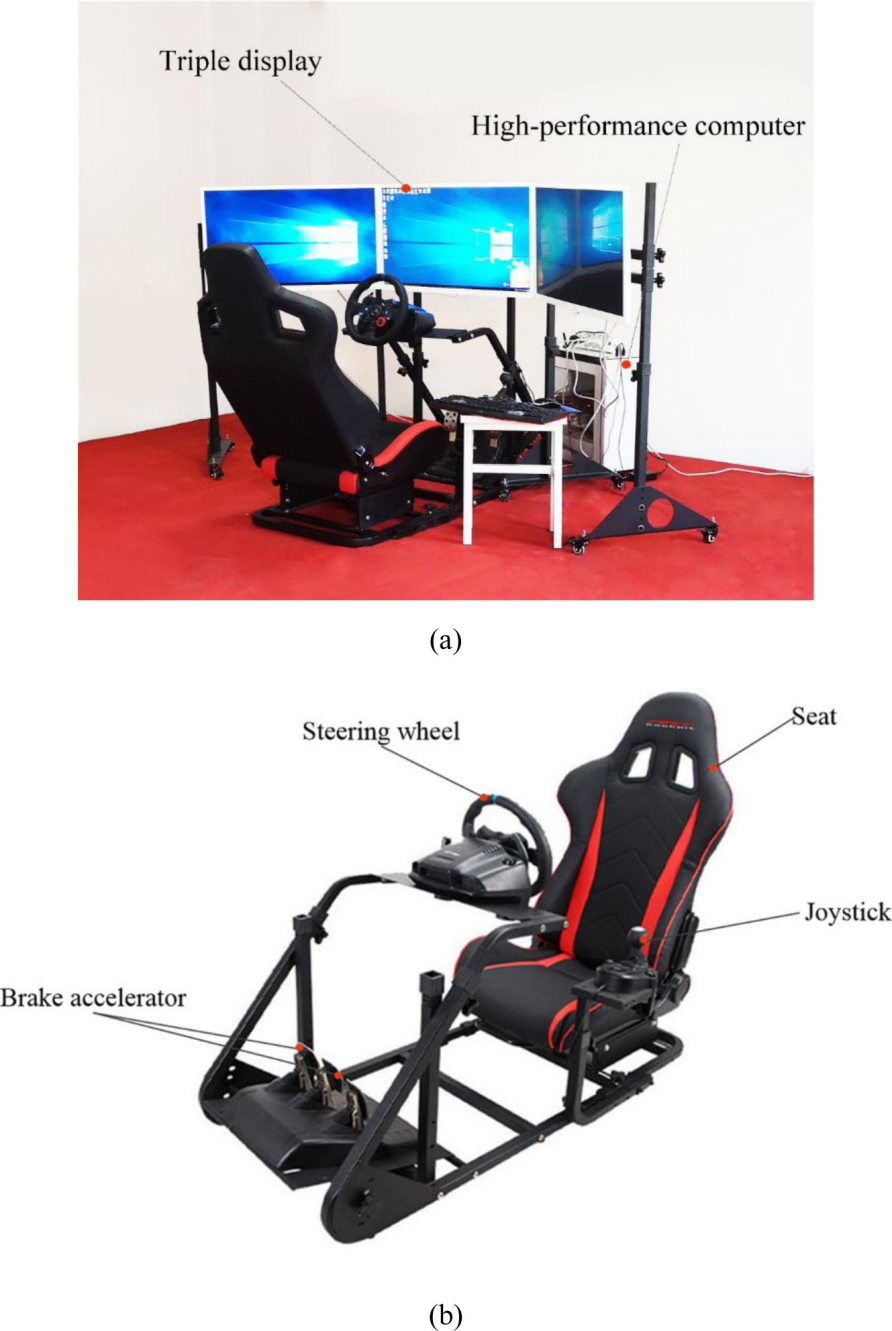
Photo of the driving simulation system setup.

Some researchers have compared results obtained using driving simulators with those obtained from field investigations and have concluded that simulator results are reliable. However, although driving simulators are an effective tool for use in analyzing the dynamic landscape [30, 32], currently available simulators are either too expensive for experiments or they do not provide suitable simulation effects. Therefore, this study develops a driving simulation system with a steering wheel, manual gears, a footrest, and a seat that also comprises a 3D computer simulation system. A detailed structural map and image of the system setup are shown in Figs 1 and 2, respectively.

The greatest advantage of this system is that automatic vehicle flow can be generated. The degree of reduction in the driving environment affects the psychology of driving, which is directly related to the value of the experimental data. Therefore, the system can better restore real driving psychological feelings.

### 2.1 Software development process

**Fig 3:**
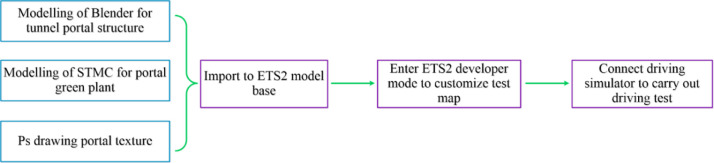
Main functions of various software applications in the driving simulation tests and development process.

ETS2 is robust gaming software that contains vivid landscape pictures and real sound effects. Furthermore, it can be connected to driving simulation equipment to provide a realistic driving experience. All software supporting the driving simulation tests are connected to ETS2, and the software function allocation and development process are shown in Fig 3. The connections between each software application were developed and studied, and the map was finally customized using ETS2 to realize driving simulation tests.

### 2.2 Construction of the driving simulation platform in Euro Truck Simulator 2

**Fig 4:**
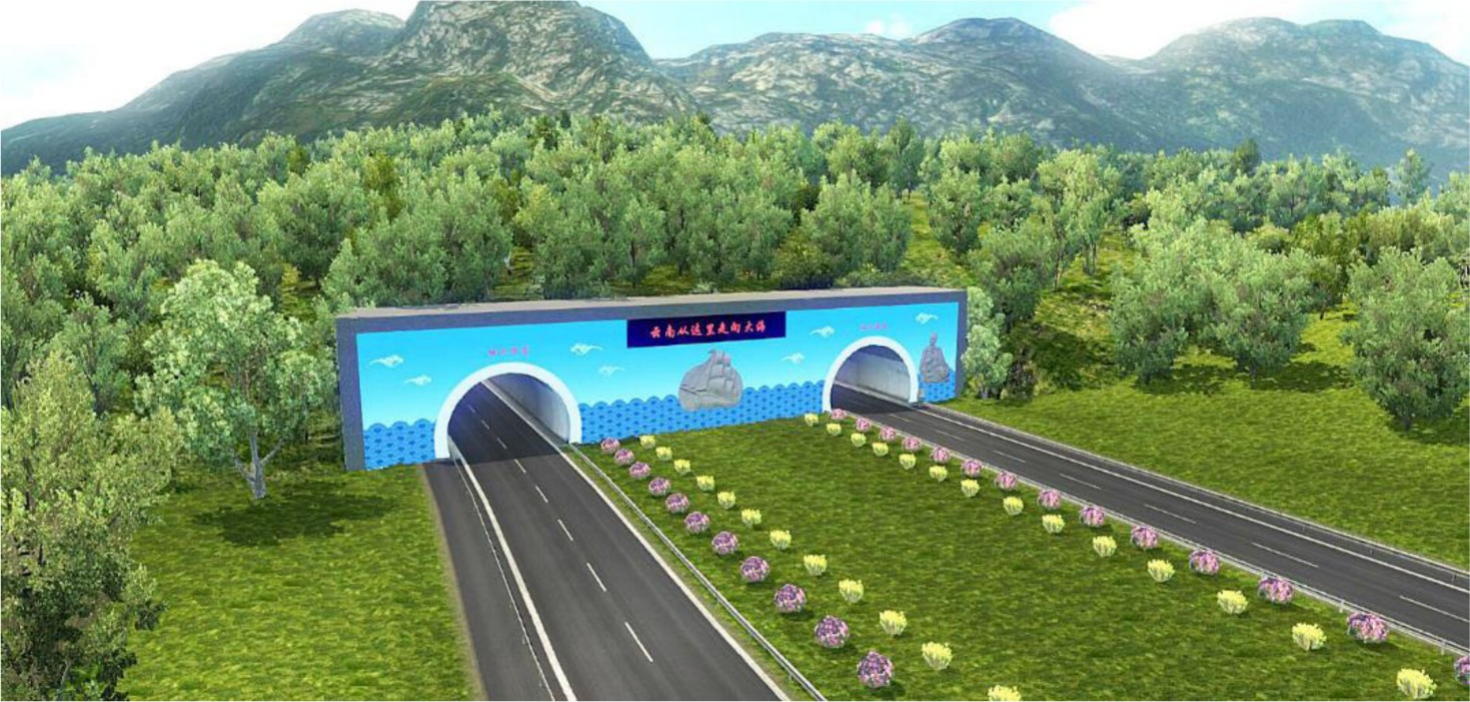
A modeling effect in ETS2.

ETS2 is also semi-open-source software, and it allows users to customize the map that is the core of the driving simulation system. This has two functions: first, establishing the map, which enables roads and mountains to be designed based on 3D coordinates extracted from Google Earth; and second, integrating the models established with other software into the platform to construct the driving simulation scene, thereby achieving a virtual reality. Participants can then evaluate and score the esthetics of a tunnel portal based on the dynamic visual effects they experience when passing through the tunnel portal. An image of the modeling effect is shown in Fig 4.

### 2.3 Connection between Blender and ETS2

**Fig 5:**
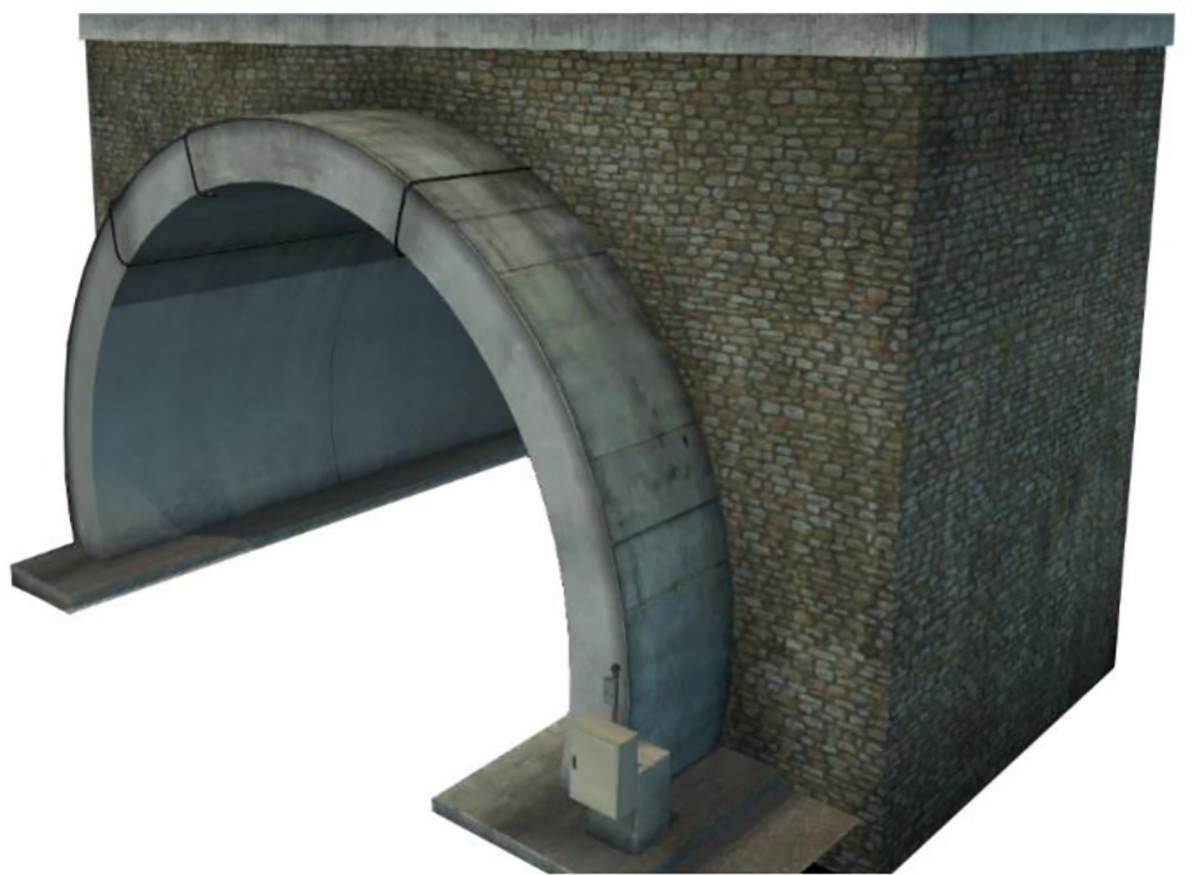
Modeling effect created with Blender.

Blender is a 3D modeling software created by a Ton Roosendaal in 1995. It integrates modeling, UV mapping, chartlets, rendering, and other functions and has the primary function of modeling in the developed driving simulation system. The use of the scs_extractor and converter_pix plug-ins to construct the interface between Blender and EST2 enables data to be imported between two software models through the development of a model definition code. Images of the detailed modeling effect are shown in Fig 5.

### 2.4 Connection between Adobe Photoshop CS6 and Blender

**Fig 6:**
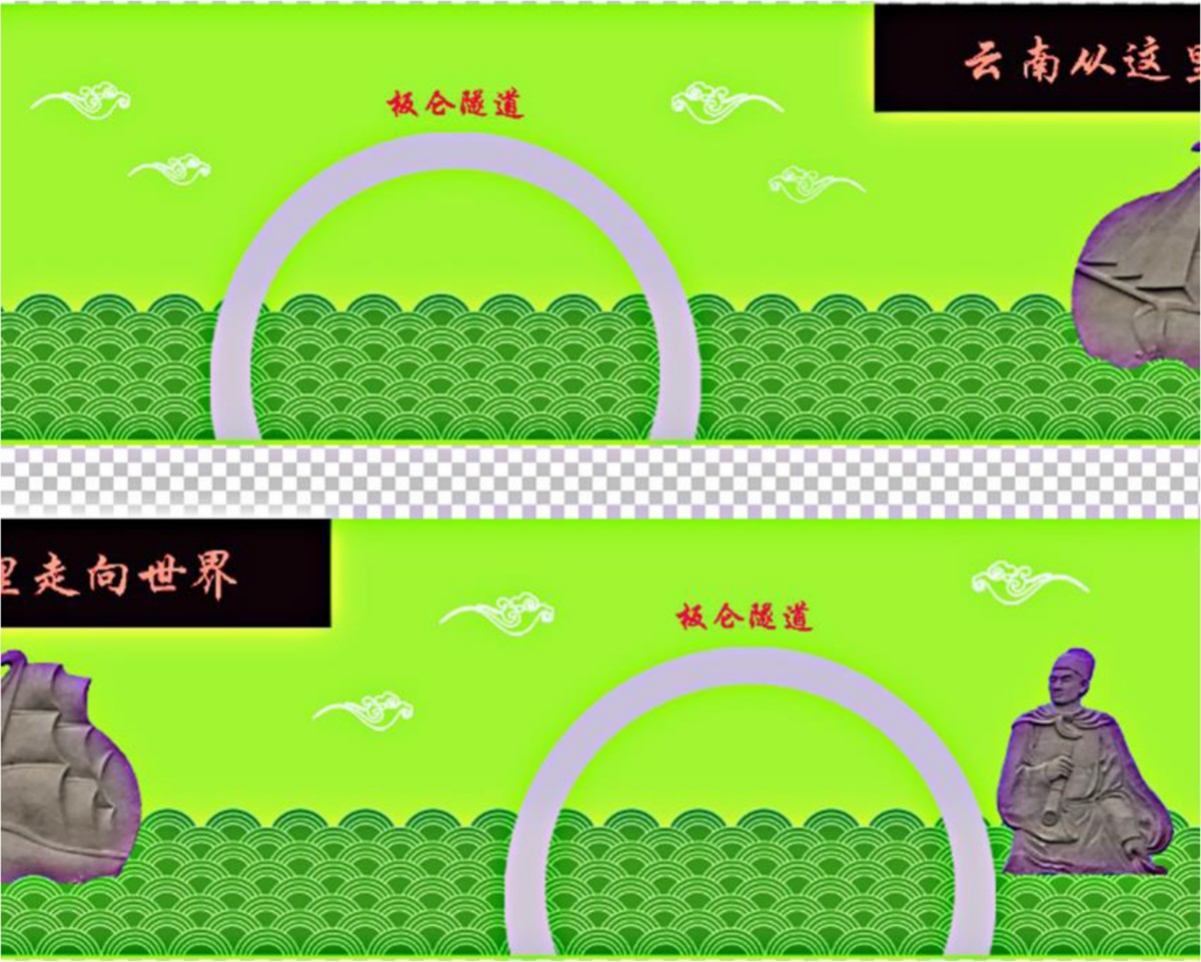
Decorative map of the tunnel portal in Ps.

**Fig 7:**
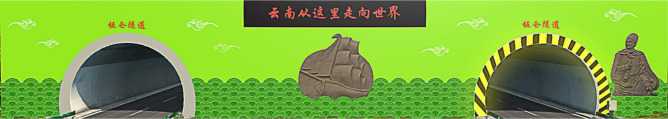
Decorative model of the portal completed in Blender (the slogan means: Yunnan is approaching the world).

Ps is a top-of-the-line image design and production software tool. The driving simulation test system developed in this study relies on Ps to provide the model with texture, color, and other attributes, as shown in Fig 6. This is achieved by using the UV mapping plate in Blender to realize UV spreading in the model and then exporting PNG files to Ps for decoration; the resulting TGA files are finally exported to Blender to complement the decoration for the portal. Fig 7 shows an image of the resulting effect.

### 2.5 Connection between SpeedTree Modeler Cinema and Blender

**Fig 8:**
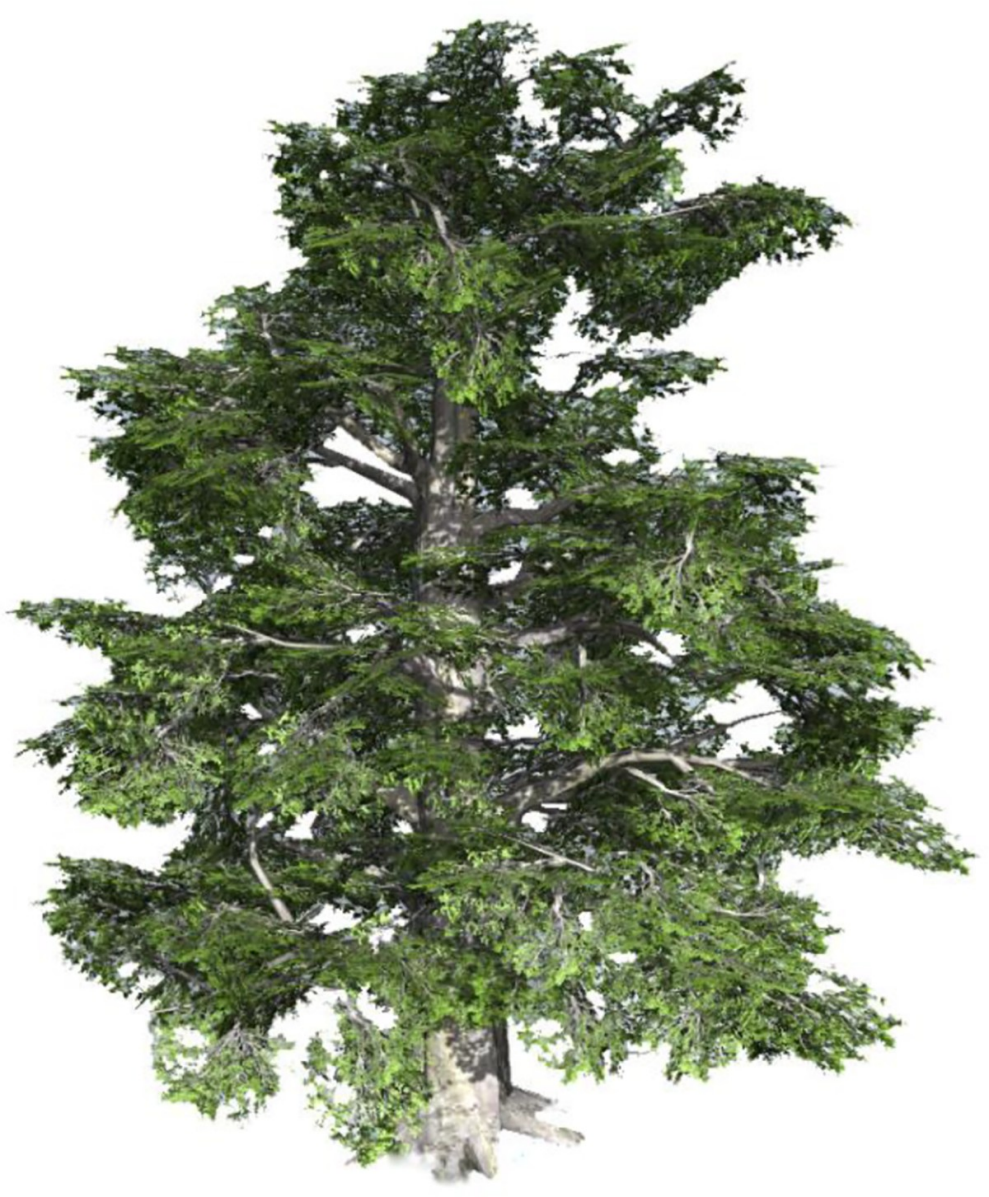
Effect of STMC landscape plant modeling.

STMC is 3D plant modeling software developed and manufactured by the award-winning US company IDV. With a physically based rendering (PBR) workflow, the software includes brand-new plant model libraries for 152 plant types, which enables the rapid design and rendering of large tracts of forest. The main function of STMC in the driving simulation system is to provide landscape plant models. This is achieved by exporting FBX files to a modeling plate through STMC for modeling. Fig 8 shows the effect of the plant modeling.

The models developed with the software presented above are then sent to the ETS2 model library through Blender. By entering ETS2 developer mode, the models are placed in accordance with 3D coordinates extracted from Google Earth to finish the design of the test scenarios.

The main advantage of our self-developed driving simulation test system is that it provides extremely delicate images, and the sunlight and shadow effects are outstanding; therefore, together with the vivid texture, simulations of the cab and landscape are excellent. During the test, the driving simulation system automatically generates the car flow to simulate real road conditions. All the sound effects in the system are recorded in a professional recording studio, and the sound of the wind and the visualization of insects during driving assist in providing a realistic driving experience. The simulation platform in the test system is semi-open-source software; hence, it is possible to independently build various different plant landscape models and structural models. Furthermore, the use of all this software is free, making it economically suitable for use in numerous experiments.

## 3. Driving simulation tests for esthetic evaluation of a tunnel portal

**Fig 9:**
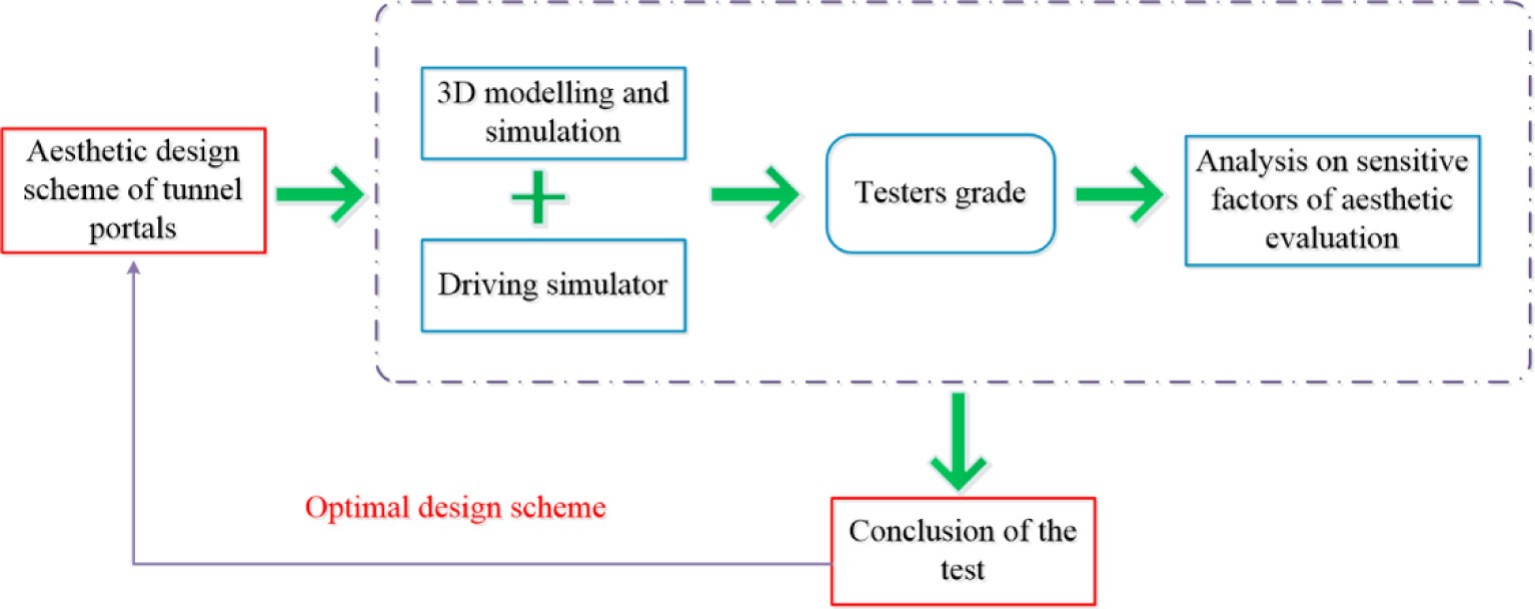
Overall concept of the driving simulation tests for esthetic evaluation of a tunnel portal.

The driving simulation tests for the esthetic evaluation of a tunnel portal in this study employ modeling software to realize 3D visualization of the structure and the landscape of the tunnel (with a driving simulator connected externally to create a virtual reality driving simulation system), as previously stated. Participants grade the tunnel landscapes when passing through the tunnel portal according to their personal preference; the optimal scheme is then chosen based on all the scores, and this is fed back to produce the design. The flow chart in Fig 9 shows the overall concept used in conducting the tests.

The tests in this study are based on the portal of the Banlun Tunnel on the Funing–Longliu Expressway. The Banlun Tunnel is a vital route connecting Yunnan and Guangxi Provinces, and it also provides the most straightforward southwest access to the sea in Yunnan Province; therefore, both the expressway and the tunnel need to provide an optimum driving experience. The tunnel is 1977.5 m long, and the portal is of a headwall type. The tunnel entrance direction is southwest, and the headwall is 59.85 m long and 13.25 m high, while the portal is 11 m wide and 8.55 m high. The driving simulation test uses the design scheme of the tunnel portal as a benchmark. In the esthetic design of a highway tunnel, both the portal and the external tunnel environment play the most important roles. When designing the impact factors, we consulted pictures of existing esthetic tunnel designs and selected the linearity, color, and texture of the portal that play an important role in portal design and the planting scheme (greening) that plays an important role when designing the outside environment.

### 3.1 Objectives of the tests

1. Esthetic evaluation of tunnel portal
2. Investigation of sensitivity to esthetic factors of tunnel portal
3. Establishment of an optimal collocation scheme for different landscape factors

### 3.2 Testing method

The esthetic effect of a tunnel portal is the result of multiple factors. Therefore, a multifactor analysis can be used to analyze the impact on results from employing and varying multiple factors. Furthermore, the influence of errors can be reduced to a certain extent. However, it is not possible to conduct comprehensive tests for multiple factors belonging to a factor set because employing such a large number of tests is impractical. Therefore, an orthogonal test is adopted when designing the driving simulation test [33, 34].

### 3.3 Evaluation subjects

Jensen investigated differences between the esthetic attitudes of different types of participants conducting evaluations, and the results indicated that landscape evaluation results are highly consistent [35]. Meanwhile, Schroeder and Daniel noted that there were marginal differences between the esthetic attitudes of student participants and the general public [36], which suggests that the esthetic attitudes of students can effectively represent those of the public. For convenience, in this experiment, 10 male graduate students were selected as the evaluation subjects: they were aged between 23 and 25, and all had more than three years of driving experience.

The study was approved by the Academic Committee of the Highway School at Chang'an University. Before implementing the study, our research plan was discussed by several experts. They believed that the driving simulation test would not cause any mental injury to the participants, nor would it have any negative social impact or affect the participants. As a consequence, they agreed that the research plan was scientifically sound and feasible, and comply with laws and regulations in China. Informed consent was obtained from each participant, and there were no minors among the participants.

### 3.4 Test procedure

#### 3.4.1 Design of orthogonal table

The object of this test was the headwall tunnel portal, which has a relatively larger area than other tunnels. Therefore, during the driving process, the color and texture of the portal were the main information elements in the driver's vision that would have the greatest influence. Meanwhile, portal greening was an important factor in reducing the difference between the tunnel portal and the surrounding environment, which determined the advantages and disadvantages of the tunnel portal environment. Therefore, four major factors were selected as sensitivity factors: linearity, color, greening, and texture of the portal.

Three levels were defined for each factor, and Table 1 summarizes the design of the orthogonal factor table. Considering the complexity of the design, it was impossible to specify the design scheme of tunnel portal greening. Moreover, when driving at a high speed, tunnel portal greening mainly existed in the driver's side view area, which could only leave a vague scene for the driver. Therefore, we chose "no greening, simple greening and delicate greening" to represent three levels of design.

**Table 1:** Defined levels of different factors for orthogonal analysis.

Factor Level	A Portal linearity	B Portal color	C Portal greening	D Portal texture
1	Straight line	Blue	Delicate greening	Mural
2	Step type	Green	Simple greening	Simple treatment
3	Arch	Concrete	None	None

Table 2 shows nine test schemes obtained from orthogonal tests. Although some of them seem not so beautiful, to ensure the balance of the tests and to further analyze the esthetic factors, we need to conduct a three-dimensional simulation and driving test analysis for each scheme. It is of note that the nine tests listed in Table 2 are conducted under nine working conditions. As the tests for each working condition required public participation, multiple tests were conducted for each working condition.

**Table 2:** *L*_9_(3^4^) orthogonal table.

Variable factor No. of test model	A Portal linearity	B Portal color	C Portal greening	D Portal texture
1	1 (Straight line)	1 (Blue)	1 (Delicate greening)	1 (Mural decoration)
2	1	2 (Green)	2 (Simple greening)	2 (Simple treatment)
3	1	3 (Concrete)	3 (None)	3 (None)
4	2 (Step type)	1	2	3
5	2	2	3	1
6	2	3	1	2
7	3 (Arch)	1	3	2
8	3	2	1	3
9	3	3	2	1

In the esthetic evaluation of the tunnel portal landscape, this paper mainly analyzes the process of tunnel portal landscape from first appearance in the drivers' vision to its disappearance (the moment of entering the tunnel entrance). Since the process of the driver entering the tunnel from the outside is not included, there is no need to consider the brightness changes inside and outside the tunnel. The brightness of the entrance landscape also has an influence on the esthetic evaluation. In the test design, the difference in brightness between colors, textures of the portal decoration and various kinds of plants will give the tester different feelings. This is also true in reality, so the test can better simulate the real situation. During the testing process, we adjusted the time to 10 o'clock in the test, reducing the interference of natural light on landscape brightness. Under the interference of the brightness of the landscape, the overall visual perception of the esthetics of the tunnel portal is mainly studied during the test.

#### 3.4.2 Construction of 3D models

**Fig 10:**
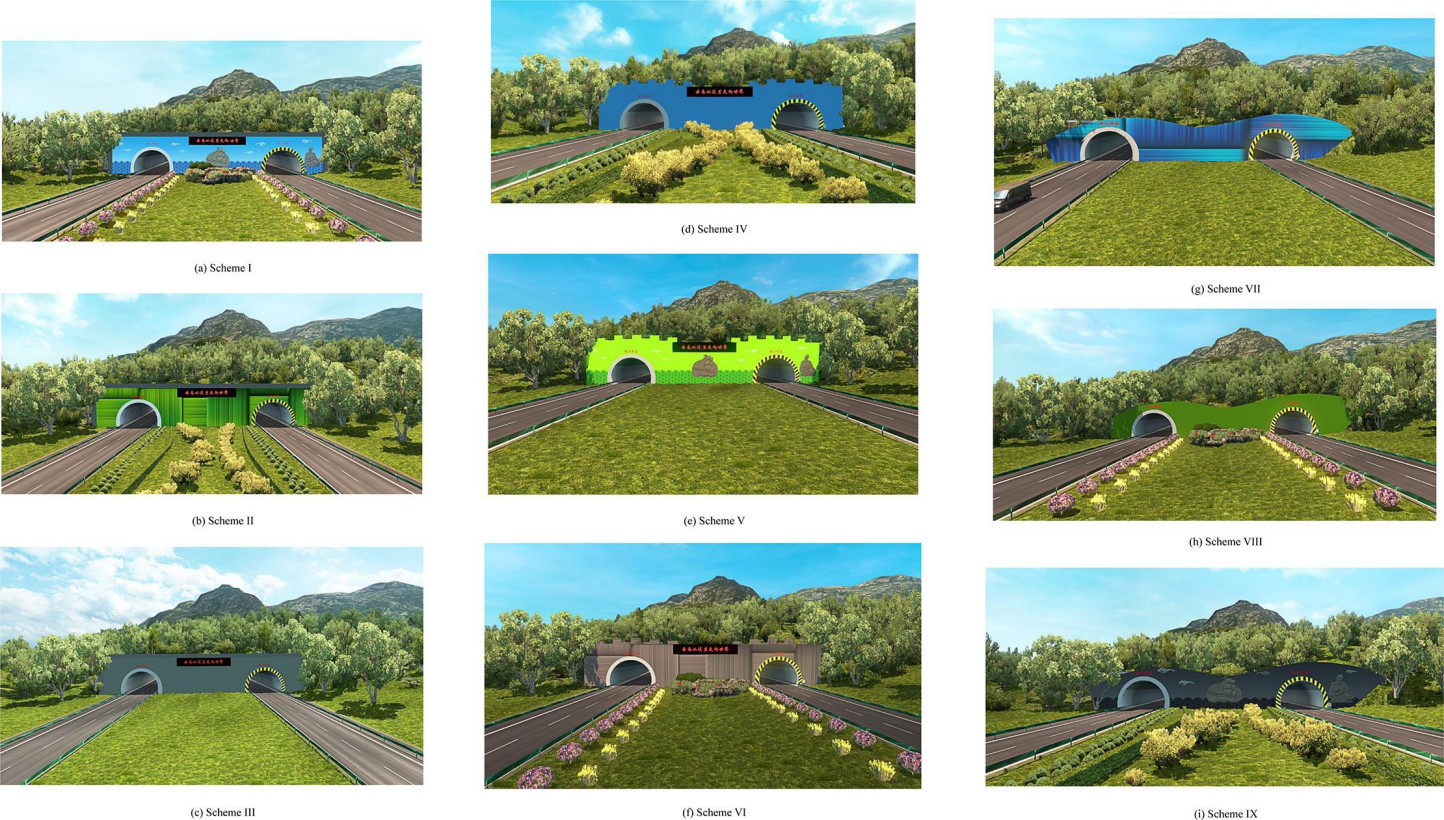
Modeling of the nine test schemes described in the *L*_9_(3^4^) orthogonal table.

Software (Blender, Ps, and STMC) was used to build the corresponding 3D models based on the orthogonal test design. The software was then imported to ETS2 to establish terrain models and to finally create the simulation scenarios for the various test schemes. Fig 10 shows the modeling effects used in the various test schemes. During modeling, the length and height of the portal, mural decoration, greening decoration and other design parameters strictly refer to the actual size.

#### 3.4.3 Test of esthetic attitude of the public

Before the test, the maximum driving speed was limited to 80 km/h through the system setting. Based on safe driving, the speed of the drivers was not required. Drivers were free to drive according to their own habits. In this way, drivers could reduce their attention to the driving speed and pay more attention to the tunnel portal landscape, thus increasing the reliability of esthetic evaluation.

Ten experienced drivers were recruited as participants. These individuals drove the simulation vehicle through the tunnel portal and then esthetically graded the tunnels. They were prohibited from discussing the tests with each other and were asked to grade the esthetics of the test samples quickly after first sight without reflecting. Fuzzy evaluation was adopted for the grading, where the participants graded their impressions of the tunnels during the driving simulation using a comment set and selecting either “great, good, ordinary, relatively bad, bad” [37–39].

**Fig 11:**
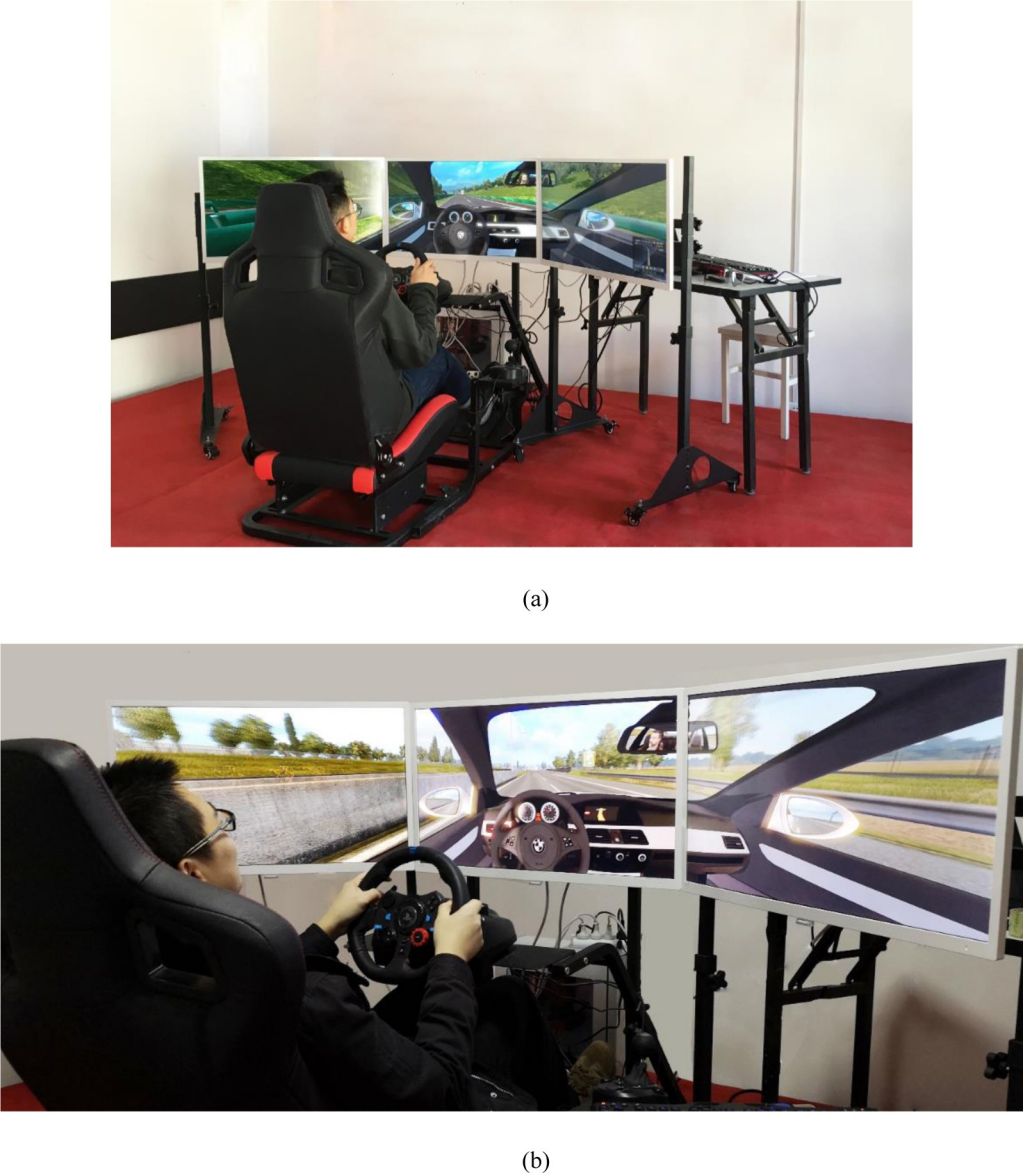
Driving simulation test process for the esthetic evaluation of a tunnel portal.

The driving tests followed a strict protocol. Prior to the test, the drivers were given time to become familiar with driving simulator operation, and during the test, the drivers stopped to grade and rest for approximately 10 min after passing through each tunnel portal prior to conducting the second driving test. Fig 11 shows the testing process.

### 3.5 Analysis of test results

To improve the reliability of the esthetic evaluation of tunnel portals, we required drivers to conduct two tests on the same test scheme. In the first test, the drivers evaluated the tunnel portal design according to their driving experience. In the second test, the drivers were familiar with the nine test plans and made judgments again.

#### 3.5.1 Esthetic evaluation and analysis of tunnel portals

The test evaluation results are shown in Table 3.

**Table 3:** Esthetic evaluation summary of nine test schemes.

	great	good	ordinary	relatively bad	bad
Scheme I	13	5	2	0	0
Scheme II	0	8	7	3	2
Scheme III	0	5	12	3	0
Scheme IV	2	9	7	2	0
Scheme V	3	8	7	2	0
Scheme VI	8	4	6	2	0
Scheme VII	4	2	10	4	0
Scheme VIII	1	7	7	5	0
Scheme IX	8	7	5	0	0

According to the fuzzy math method, taking Scheme I as an example, the calculation process of the esthetic evaluation results of the tunnel portal is illustrated:

Normalizing the numerical value of the Scheme I tunnel portal esthetic evaluation set, the membership matrix R is obtained as follows:

R={0.65,0.25,0.1,0,0} (1)

Evaluation set E = {e_1_, e_2_, e_3_, e_4_, e_5_} = {great, good, ordinary, relatively bad, bad} = {95, 85, 75, 65, 60}; then, the calculation method of the Scheme I tunnel portal esthetics evaluation value is as follows:

$$S_{\mathrm{beautify}}=ER^{T}=[95,85,75,65,60]\times \left[\begin{array}{c} 0.65\\ 0.25\\ 0.1\\ 0\\ 0 \end{array}\right]=90.5$$ (2)

The calculation method of the esthetic score of other schemes is the same and will not be discussed in detail here. Finally, the esthetic evaluation values of the nine schemes are shown in Table 4.

**Table 4:** Summary of esthetic evaluation results for nine experimental schemes.

	Scheme I	Scheme II	Scheme III	Scheme IV	Scheme V	Scheme VI	Scheme VII	Scheme VIII	Scheme IX
The beauty of score	90.5	76	76	80.5	81	84	78	77	86.5

Through the analysis results of the above nine design schemes, Scheme I scored the highest (90.5), and its beauty was deemed the best. Scheme II and Scheme III scored the lowest, and their beauty was deemed the worst.

#### 3.5.2 Analysis of sensitivity factors of tunnel portal esthetic

To guide the design effectively, the influencing factors of tunnel portal esthetics are sorted by means of the range analysis method in orthogonal tests, that is, an esthetic sensitivity factor analysis of the tunnel portal is carried out.

In a well-designed orthogonal table, if we assume that the sum of the results of level i for the factors in column j is *k*_*ji*_, then ${\bar{k}}_{ji}$ is the mean value of *k*_*ji*_, and *R*_*j*_ is the range of factors in column j (i.e., the difference between the maximum and minimum index values obtained from all levels of the factors in column j) as follows:

$$R_{j}=\max\left\{{\bar{k}}_{j1},{\bar{k}}_{j2},\cdots ,{\bar{k}}_{ji}\right\}-\min\left\{{\bar{k}}_{j1},{\bar{k}}_{j2},\cdots ,{\bar{k}}_{ji}\right\}$$ (3)

When *R*_*j*_ has a larger value, the impact of that factor on the test result is also large. The magnitude of importance and incidence to the test results for all factors in the design are confirmed based on the ordering of *R*_*j*_ by magnitude. In addition, the best collocation design scheme can be chosen based on an analysis of *k*_*ij*_. This method was used in this study to analyze the data.

Based on the test results, the counting process used for the range of analysis data is summarized in Table 5.

**Table 5:** Range analysis calculation results.

Test number	A Portal linear	B Portal color	C Portal greening	D Portal texture	Esthetics score E (mean value)
	1	2	3	4	${\bar{y}}_{i}$
1	1 (Straight line)	1 (Blue)	1 (Delicate greening)	1 (Mural)	90.5
2	1	2 (Green)	2 (Simple greening)	2 (Simple greening)	76
3	1	3 (Concrete)	3 (None)	3 (None)	76
4	2 (Step type)	1	2	3	80.5
5	2	2	3	1	81
6	2	3	1	2	84
7	3 (Arch)	1	3	2	78
8	3	2	1	3	77
9	3	3	2	1	86.5
*K*_*j*1_	242.5	249	251.5	258	
*K*_*j*2_	245.5	234	243	238	
*K*_*j*3_	241.5	246.5	235	233.5	
${\bar{K}}_{j1}$	80.833	83.000	83.833	86.000	
${\bar{K}}_{j2}$	81.833	78.000	81.000	79.333	
${\bar{K}}_{j3}$	80.500	82.167	78.333	77.833	
R_*j*_	1.333	5.000	5.500	8.167	

The following conclusions were drawn by employing the method used to treat and analyze the above data:

1. The importance of factors can be sorted in descending order based on the magnitude of the range, *R*_*j*_, as follows: the portal texture exerts the maximum impact on the beauty degree of the headwall portal, followed by the portal greening and the portal color, while the portal linearity exerts the minimum impact;
2. Based on the principle relating to esthetic scores (a higher esthetic score is better), corresponding levels for the maximum {*k*_*j*1_,*k*_*j*2_,*k*_*j*3_} can be chosen for each factor as the highest level. Based on this, the optimal scheme in these tests is A_2_B_1_C_1_D_1_.

## 4. Conclusion and prospects

Rapid developments in landscape construction within China in recent years have led to the need to ensure that structures are designed with a consideration of esthetics. A complete and scientific esthetic evaluation methodology is an effective way of providing a balanced esthetic design. Through a series of orthogonal tests conducted using a self-developed driving simulation test system, this study performed an experimental investigation evaluating the esthetics of the Banlun Tunnel portal. The following conclusions can be drawn:

1. Based on the driving simulation platform Euro Truck Simulator 2, we combined this platform with Blender, SpeedTree Modeler Cinema, Adobe Photoshop CS6 and other software for secondary development and built a driving simulation test system equipped with a driving simulator. The system can restore the driving process well and evaluate the esthetics of the tunnel portal from a dynamic perspective, which not only improves the reliability of the test evaluation results but also serves as a new way to explore dynamic landscape design.
2. The esthetic score of the tunnel entrance in the driving simulation test is calculated by the fuzzy mathematics method, and the esthetic sensitivity factor of the tunnel entrance is analyzed by the range analysis method in an orthogonal test. The order of participants’ sensitivity to esthetic factors for the headwall portal in descending order is listed as follows: the portal texture exerts the maximum impact on the beauty degree of the headwall portal, followed by the portal greening and the portal color, while the portal linearity exerts the minimum impact.
3. Of the nine design schemes proposed for the Banlun Tunnel portal, scheme І was considered the most esthetically pleasing. The optimal scheme was determined as A_2_B_1_C_1_D_1_, in which the portal linearity is step-type, the portal is decorated with a blue mural, and delicate greening surrounding the portal.

Tunnel portal has always been a black spot. In the absence of esthetic design criteria for tunnel entrances, designers may design overly attractive tunnel portal landscapes, which will attract too much attention from drivers and introduce hidden dangers to traffic safety in the tunnel entrance landscape. In a follow-up study, we will conduct future research on the safety of tunnel entrances to create a beautiful, comfortable and safe tunnel portal environment for drivers.

## Supporting information

S1 Dataset (XLSX)
